# On the use of absolute interface coordinates in the floating frame of reference formulation for flexible multibody dynamics

**DOI:** 10.1007/s11044-017-9606-3

**Published:** 2017-12-14

**Authors:** Marcel Ellenbroek, Jurnan Schilder

**Affiliations:** Faculty of Engineering Technology, University of Twente, P.O. Box 217, 7500AE Enschede, The Netherlands

**Keywords:** Flexible multibody dynamics, Floating frame formulation, Corotational frame formulation, Inertial frame formulation, Craig–Bampton method, Model order reduction

## Abstract

In this work a new formulation for flexible multibody systems is presented based on the floating frame formulation. In this method, the absolute interface coordinates are used as degrees of freedom. To this end, a coordinate transformation is established from the absolute floating frame coordinates and the local interface coordinates to the absolute interface coordinates. This is done by assuming linear theory of elasticity for a body’s local elastic deformation and by using the Craig–Bampton interface modes as local shape functions. In order to put this new method into perspective, relevant relations between inertial frame, corotational frame and floating frame formulations are explained. As such, this work provides a clear overview of how these three well-known and apparently different flexible multibody methods are related. An advantage of the method presented in this work is that the resulting equations of motion are of the differential rather than the differential-algebraic type. At the same time, it is possible to use well-developed model order reduction techniques on the flexible bodies locally. Hence, the method can be employed to construct superelements from arbitrarily shaped three dimensional elastic bodies, which can be used in a flexible multibody dynamics simulation. The method is validated by simulating the static and dynamic behavior of a number of flexible systems.

## Introduction

Flexible multibody dynamics is concerned with the study of machines and mechanisms that consist of multiple deformable bodies. Although the elastic deformation within a single body can often be considered as small, the large rigid body rotations between different bodies make the problem to be of a geometrically nonlinear nature.

The kinematics of a body can be described by the motion of a set of coordinate frames, each frame being rigidly attached to a material point on the body. Connections between bodies are introduced by kinematic constraints. These relate the motions of the coordinate frames of the so-called interface points located at different bodies.

**Fig. 1:**
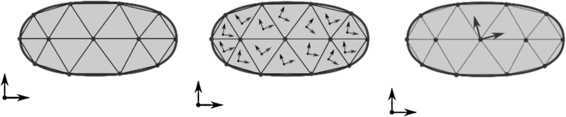
Graphical representation of a single flexible body using three different formulations. The inertial frame formulation is expressed in the absolute nodal coordinates (*left*). The corotational formulation uses a corotational frame for each element (*center*). The floating frame formulation uses a floating frame for each body (*right*). Figure was made using InkScape

Three essentially different commonly used descriptions are available for flexible multibody systems: the inertial frame formulations, the corotational frame formulations and the floating frame formulations. These formulations are significantly different in the way they describe a body’s elastic behavior. Hence, there are important differences in the choice of the degrees of freedom and consequently in the way kinematic constraints between bodies are enforced. Figure 1 gives a graphical interpretation of each formulation. A comprehensive overview of these formulations, their background and particularities can be found in [1].

The inertial frame formulation is essentially a nonlinear finite element formulation of which the details can be found in standard textbooks, such as [2]. Elastic deformations are described using the nonlinear Green–Lagrange strain definition, which describes large rigid body rotations correctly. In this formulation, each body is discretized using a set of global interpolation functions. The degrees of freedom are the absolute positions and orientations of the coordinate frames located at the nodes of the finite element mesh. Constraints between bodies can be simply enforced by directly equating the appropriate degrees of freedom of the nodes that both bodies have in common at a certain interface point. For applications to flexible multibody systems, the geometrically exact beam theory is a commonly used example of an inertial frame formulation [3]. However, difficulties in frame invariance may arise due to the interpolation of the large rotations [4, 5].

The corotational frame formulation can be interpreted as the geometrically nonlinear extension of a linear finite element formulation [6, 7]. A corotational frame describes the large rigid body motion of an element with respect to the inertial frame. Small elastic deformations within the element are superimposed using the linear finite element matrices, based on the Cauchy strain definition. The nonlinear finite element model is obtained from the linear finite element model by simply pre- and post-multiplying the mass and stiffness matrices with the rotation matrices corresponding to the corotational frames. Note that the formulation is still able to geometrically describe geometrical nonlinear elastic deformations within a single body due to the fact that each element is given its own corotational frame. That is, provided that the small strain assumption within an element holds.

Similar to the inertial frame formulation, the corotational frame formulation uses the absolute nodal coordinates as degrees of freedom. Consequently, both methods also enforce constrains in the same way. However, in order to arrive at this formulation, a unique kinematic relation must be established that expresses the coordinates of an element’s corotational frame in terms of the element’s absolute nodal coordinates. For a variety of standard elements, this is commonly done by locating the corotational frame in one of the element’s nodes or by expressing the corotational frame coordinates as a certain weighted average of the element’s nodal coordinates. Alternatively, it is possible to define the orientation of each corotational frame by demanding zero elastic deformation at its location [8]. A drawback of standard corotational frame formulations is that they do not distinguish between rigid and flexible bodies. That is, rigid bodies are modeled as flexible bodies with a large stiffness, resulting in a less efficient formulation for multibody systems that contain both rigid and flexible bodies.

The floating frame formulation can be interpreted as the flexible extension of a rigid multibody formulation. A floating frame describes the large rigid body motion of a body with respect to the inertial frame. When linear theory of elasticity is used to describe the flexible behavior locally, mass and stiffness matrices are obtained from a body’s linear finite element model. These system matrices can be reduced using a wide variety of comprehensive well-developed model order reduction techniques, such as the Craig–Bampton method [9, 10].

In the floating frame formulation, the degrees of freedom thus consist of the absolute coordinates of the floating frame and a set of local generalized coordinates used to describe the body’s local linear elastic behavior. Because the absolute interface coordinates are not part of the degrees of freedom, the kinematic constraints are typically highly nonlinear equations in terms of many degrees of freedom. As no analytical solution of these equations might be found, the constraints are commonly enforced using Lagrange multipliers, increasing the total number of unknowns. The resulting equations of motion are combined differential-algebraic equations [11, 12].

The possibility of using well-developed model order reduction techniques to reduce computational cost makes the floating frame formulations the preferred formulation in many situations in which the elastic deformation within a body can be considered small. The disadvantage of this formulation in satisfying kinematic constraints could be eliminated if it is possible to express the floating frame coordinates and the local elastic deformation directly in terms of the interface coordinates. The search for such a kinematic coordinate transformation has led to the development of superelements in flexible multibody formulations. Methods have been developed in which the floating frame is located at an interface point [13] or expressed as the weighted average of the interface coordinates [14]. The first option introduces an unwanted discrimination between the interface points, which makes the results dependent on the interface point chosen. Moreover, in general a better accuracy is obtained when the floating frame is close to the body’s center of mass. In the second option the motion of the floating frame cannot be interpreted as the motion of a material point, but only as the body’s average rigid body motion.

In this paper, a new method is presented, with which it is possible to obtain a floating frame formulation in terms of interface coordinates only, that does not suffer from the disadvantages mentioned above. The method offers the combined advantage of being able to enforce constraints without the use of Lagrange multipliers and still have the possibility to use Craig–Bampton based model order reduction techniques on the bodies’ linear finite element models. In fact, the method demonstrates the interchangeability of the absolute interface coordinates and the combination of the floating frame coordinates and local elastic coordinates corresponding to these Craig–Bampton modes. It will be shown that in the dynamic equations local coordinates can be substituted by global coordinates, and vice versa.

Essential for the method presented here is the fact that the Craig–Bampton modes are able to describe rigid body motions. In the floating frame formulation, these rigid body modes must be eliminated in order to describe the system’s motion uniquely. However, in this work this property of the Craig–Bampton modes is used to eliminate the floating frame coordinates and express the local elastic degrees of freedom solely in terms of the global motion of the interface points. This is done by demanding that the elastic body has no deformation at the location of the floating frame. This requirement is met without the need to locate the floating frame in an interface point.

The subsequent sections are organized as follows: In Sect. 2, the kinematic description of a coordinate system attached to a material point of a flexible body is introduced using a position vector and a rotation matrix. In Sect. 3, the kinematics of the floating frame formulation are introduced. A relation is developed that expresses the local coordinates of a material point on a flexible body as the difference between the absolute coordinates of that material point and the floating frame coordinates. Section 4 introduces the static Craig–Bampton modes to describe a body’s local linear elastic displacement field. It is shown that the generalized coordinates corresponding to these modes can be expressed in terms of the absolute interface coordinates and the floating frame coordinates. In Sect. 5, the floating frame coordinates are expressed in terms of the absolute interface coordinates by demanding zero deformation at the location of the floating frame. In Sect. 6, the obtained coordinate transformations are applied to the standard equation of motion of the floating frame formulation. At this point, the kinematic constraint equations are applied without using Lagrange multipliers. The resulting equations of motion can be solved incrementally using numerical time integration. Section 7 demonstrates the new method on a number of test cases. The most important conclusions finalize the paper.

## Kinematics of a material point on a flexible body

**Fig. 2:**
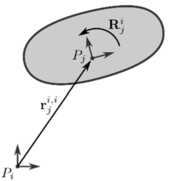
The position of $P_{j}$ with respect to $P_{i}$ using a position vector and rotation matrix. Figure was made using InkScape

Consider a flexible body with a material point $P_{j}$. A coordinate frame $E_{j}$ is rigidly attached to this material point such that the pair $\{ P_{j}, E_{j} \}$ defines a Euclidean coordinate system. As $\{ P_{j}, E_{j} \}$ defines both the location and orientation of the frame attached to $P_{j}$, the pair will be referred to as the generalized position, or simply the position of $P_{j}$. This position can be expressed relative to another position $\{ P_{i}, E_{i} \}$ by the ($3\times1$) position vector $\mathbf{r}_{j}^{i,i}$ and the ($3\times3$) rotation matrix $\mathbf{R}_{j}^{i}$. The position vector $\mathbf{r}_{j}^{i,i}$ defines the position of $P_{j}$ (lower index $j$) relative to $P_{i}$ (second upper index $i$) and its components are expressed in the coordinate system $\{ P_{i}, E_{i} \}$ (first upper index $i$). The rotation matrix $\mathbf{R}_{j}^{i}$ defines the orientation of $E_{j}$ (lower index $j$) relative to $E_{i}$ (upper index $i$) expressed in $\{ P_{i}, E_{i} \}$. Figure 2 shows a graphical representation of the position of a material point using the position vector and rotation matrix. The two notations $\{ P_{j}, E_{j} \}$ and $\{ \mathbf{r}_{j}^{i, i}, \mathbf{R}_{j}^{i} \}$ will both be used throughout this work, depending on the context, to identify a position.

$\mathbf{R}_{j}^{i}$ also defines a coordinate transformation that can be used to transform a vector that is expressed in coordinate system $j$ to a vector that is expressed in coordinate system $i$. For example, the components of the position vector of $P_{j}$ with respect to $P_{i}$ in frames $\{ P_{i}, E_{i} \}$ and $\{ P_{j}, E_{j} \}$ are related as:

2.1 $$ \mathbf{r}_{j}^{i, i} = \mathbf{R}_{j}^{i} \mathbf{r}_{j}^{j, i}, $$

where it is seen that in this notation the first upper index has changed. The rotation matrix is an orthogonal matrix of the proper kind, which means that its determinant equals $+1$ and its transpose equals its inverse, which also represents the inverse coordinate transformation such that

2.2 $$ \bigl( \mathbf{R}_{j}^{i} \bigr)^{-1} = \mathbf{R}_{i}^{j},\quad \quad \mathbf{R}_{j}^{i} \mathbf{R}_{i}^{j} = \mathbf{I}, $$

with $\mathbf{I}$ the ($3\times3$) unity matrix. Differentiating (2.2), it can be shown that the time derivative of the rotation matrix equals a skew symmetric matrix times the rotation matrix itself. This can be written as

2.3 $$ \dot{\mathbf{R}}_{j}^{i} = \widetilde{\boldsymbol{\omega}}_{j}^{i,i} \mathbf{R}_{j}^{i}, $$

in which the tilde operator is introduced such that when applied on a ($3\times1$) vector $\mathbf{a}$, it yields a skew symmetric ($3\times3$) matrix $\tilde{\mathbf{a}}$:

2.4 $$ \tilde{\mathbf{a}} = \left [ \textstyle\begin{array}{c@{\quad}c@{\quad}c} 0 & - a_{3} & a_{2}\\ a_{3} & 0 & - a_{1}\\ - a_{2} & a_{1} & 0 \end{array}\displaystyle \right ]. $$

In (2.3), the tilde operator is applied on the vector $\boldsymbol{\omega}_{j}^{i,i}$, which is the angular velocity vector of frame $\{ P_{j}, E_{j} \}$ with respect to $\{ P_{i}, E_{i} \}$ with its components expressed in $\{ P_{i}, E_{i} \}$. The linear velocity of $\{ P_{j}, E_{j} \}$ with respect to $\{ P_{i}, E_{i} \}$ expressed in $\{ P_{i}, E_{i} \}$ is simply denoted by $\dot{\mathbf{r}}_{j}^{i,i}$. The linear and angular velocities can be combined in the $( 6\times1 )$ velocity vector $\mathbf{v}_{j}^{i,i}$ as follows:

2.5 $$ \mathbf{v}_{j}^{i,i} \equiv \left [ \textstyle\begin{array}{c} \dot{\mathbf{r}}_{j}^{i,i}\\ \boldsymbol{\omega}_{j}^{i,i} \end{array}\displaystyle \right ]. $$

To make a clear distinction between the inertial reference frame and any other coordinate system, $\{ P_{O}, E_{O} \}$ is used to denote the inertial frame. In this case $\{ \mathbf{r}_{j}^{O,O}, \mathbf{R}_{j}^{O} \}$ defines the absolute position of material point $P_{j}$ and $\mathbf{v}_{j}^{O,O}$ defines its absolute velocity.

## Kinematics of a material point using the floating frame formulation

**Fig. 3:**
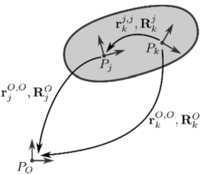
The position of material point $P_{k}$ relative to $P_{O}$ using floating frame $P_{j}$. Figure was made using InkScape

Figure 3 shows a flexible body to which a floating frame is attached in material point $P_{j}$. The absolute position of the floating frame with respect to the inertial frame located in $P_{O}$ is denoted by $\{ \mathbf{r}_{j}^{O, O}, \mathbf{R}_{j}^{O} \}$. The position of another material point $P_{k}$ relative to the floating frame is denoted by $\{ \mathbf{r}_{k}^{j, j}, \mathbf{R}_{k}^{j} \}$. For the rotation from $P_{k}$ to $P_{O}$ holds:

3.1 $$ \mathbf{R}_{k}^{O} = \mathbf{R}_{j}^{O} \mathbf{R}_{k}^{j}. $$

The position of $P_{k}$ in the inertial frame can be expressed in terms of its relative position and the absolute position of the floating frame as

3.2 $$ \mathbf{r}_{k}^{O, O} = \mathbf{r}_{j}^{O, O} + \mathbf{R}_{j}^{O} \mathbf{r}_{k}^{j, j}. $$

The absolute linear velocity of $P_{k}$ in the inertial frame is found by taking the time derivative of (3.2):

3.3 $$ \dot{\mathbf{r}}_{k}^{O, O} = \dot{\mathbf{r}}_{j}^{O, O} + \widetilde{\boldsymbol{\omega}}_{j}^{O,O} \mathbf{R}_{j}^{O} \mathbf{r}_{k}^{j, j} + \mathbf{R}_{j}^{O} \dot{\mathbf{r}}_{k}^{j, j}. $$

For the absolute angular velocity of $P_{k}$ we get

3.4 $$ \boldsymbol{\omega}_{k}^{O, O} = \boldsymbol{\omega}_{j}^{O, O} + \mathbf{R}_{j}^{O} \boldsymbol{\omega}_{k}^{j, j}. $$

Equations (3.3) and (3.4) can be rewritten and combined to

3.5 $$\begin{aligned} \left [ \textstyle\begin{array}{c} \dot{\mathbf{r}}_{k}^{O,O}\\ \boldsymbol{\omega}_{k}^{O,O} \end{array}\displaystyle \right ] = \left [ \textstyle\begin{array}{c@{\quad}c} \mathbf{R}_{j}^{O} & \boldsymbol{0}\\ \boldsymbol{0} & \mathbf{R}_{j}^{O} \end{array}\displaystyle \right ] \left [ \textstyle\begin{array}{c@{\quad}c} \mathbf{I} & - \tilde{\mathbf{r}}_{k}^{j,j}\\ \boldsymbol{0} & \mathbf{I} \end{array}\displaystyle \right ] \left [ \textstyle\begin{array}{c@{\quad}c} \mathbf{R}_{O}^{j} & \boldsymbol{0}\\ \boldsymbol{0} & \mathbf{R}_{O}^{j} \end{array}\displaystyle \right ] \left [ \textstyle\begin{array}{c} \dot{\mathbf{r}}_{j}^{O,O}\\ \boldsymbol{\omega}_{j}^{O,O} \end{array}\displaystyle \right ] + \left [ \textstyle\begin{array}{c@{\quad}c} \mathbf{R}_{j}^{O} & \boldsymbol{0}\\ \boldsymbol{0} & \mathbf{R}_{j}^{O} \end{array}\displaystyle \right ] \left [ \textstyle\begin{array}{c} \dot{\mathbf{r}}_{k}^{j,j}\\ \boldsymbol{\omega}_{k}^{j,j} \end{array}\displaystyle \right ]. \end{aligned}$$

For this, several relevant properties of skew symmetric matrices are used. A convenient overview of these properties is given in [11]. Equation (3.5) can be written in compact form using the definition of the velocity vector (2.5):

3.6 $$ \mathbf{v}_{k}^{O, O} = \bigl[ \mathbf{R}_{j}^{O} \bigr] \bigl[ - \tilde{\mathbf{r}}_{k}^{j,j} \bigr] \bigl[ \mathbf{R}_{O}^{j} \bigr] \mathbf{v}_{j}^{O,O} + \bigl[ \mathbf{R}_{j}^{O} \bigr] \mathbf{v}_{k}^{j,j}. $$

Here the notations $[ \mathbf{R}_{j}^{O} ]$ and $[ - \tilde{\mathbf{r}}_{k}^{j,j} ]$ are introduced to simplify and shorten the notation. They represent the $( 6\times6 )$ compound rotation matrix and $( 6\times6 )$ compound position matrices in Eq. (3.5), respectively. The velocity $\mathbf{v}_{k}^{j,j}$ is the local velocity of $P_{k}$ caused by the elastic deformation of the body expressed in the floating frame. Equation (3.6) can be reformulated as

3.7 $$ \mathbf{v}_{k}^{j,j} = \bigl[ \mathbf{R}_{O}^{j} \bigr] \mathbf{v}_{k}^{O,O} - \bigl[ - \tilde{ \mathbf{r}}_{k}^{j,j} \bigr] \bigl[ \mathbf{R}_{O}^{j} \bigr] \mathbf{v}_{j}^{O,O}. $$

Equation (3.7) shows that the relative velocity of the elastic deformation in the floating frame is defined by the difference of the absolute velocities in $P_{k}$ and $P_{j}$.

## Relation between the local elastic velocities and absolute velocities

It is assumed that the kinematics of a flexible body in a floating frame can be described with linear finite element models. The material behavior satisfies Hooke’s law and the strains are approximated with the linear Cauchy strain tensor [15, 16]. Based on this model, the dynamic behavior of a flexible body is fully described in the floating frame $\{ P_{j}, E_{j} \}$ by a constant mass matrix $\mathbf{M}_{j}$ and stiffness matrix $\mathbf{K}_{j}$. Model order reduction techniques can be applied by assuming that the deformation of a body is a linear combination of selected modes. Here the static Craig–Bampton modes (also known as interface modes or boundary modes) will be used as a reduction basis. The generalized coordinates $\mathbf{q}_{k}^{j,j}$ corresponding with these modes are the small elastic displacements $\mathbf{u}_{k}^{j,j}$ and rotations $\boldsymbol{\theta}_{k}^{j,j}$ of the interface points $P_{k}$ on the body:

4.1 $$ \mathbf{q}_{k}^{j,j} = \left [ \textstyle\begin{array}{c} \boldsymbol{\theta}_{k}^{j,j}\\ \mathbf{u}_{k}^{j,j} \end{array}\displaystyle \right ]. $$

The position vector $\mathbf{r}_{k}^{j,j}$ is the relative location of interface point $P_{k}$ in the body reference frame $\{ P_{j}, E_{j} \}$ and can be expressed as

4.2 $$ \mathbf{r}_{k}^{j,j} = \mathbf{x}_{k}^{j,j} + \mathbf{u}_{k}^{j,j} \bigl( \mathbf{x}_{k}^{j,j} \bigr), $$

where the position vector $\mathbf{x}_{k}^{j,j}$ is the location of $P_{k}$ on the undeformed body, which is constant. Using (2.2) in rewriting (4.2), the elastic displacement $\mathbf{u}_{k}^{j,j}$ can be expressed in terms of the absolute position of $P_{k}$ and the absolute position of the floating frame $P_{j}$:

4.3 $$ \mathbf{u}_{k}^{j,j} = \mathbf{r}_{k}^{j,j} - \mathbf{x}_{k}^{j,j} = \mathbf{R}_{O}^{j} \bigl( \mathbf{r}_{k}^{O,O} - \mathbf{r}_{j}^{O,O} \bigr) - \mathbf{x}_{k}^{j,j}. $$

For small deformations, the rotation matrix $\mathbf{R}_{k}^{j}$ can be related to the nodal rotations $\boldsymbol{\theta}_{k}^{j,j}$ directly, since within the framework of linear theory all parameterizations of $\mathbf{R}_{k}^{j}$ yield the same results. To this end, consider the Taylor expansion of $\mathbf{R}_{k}^{j}$ at $t = t_{0} +\Delta t$ around the undeformed configuration and use the exponential map [17] to approximate the rotation matrix as:

4.4 $$ \mathbf{R}_{k}^{j} \bigl( \boldsymbol{\theta}_{k}^{j, j} +\Delta t \boldsymbol{\omega}_{k}^{j, j} \bigr) = \exp \bigl( \widetilde{\boldsymbol{\theta}}_{k}^{j, j} +\Delta t \widetilde{\boldsymbol{\omega}}_{k}^{j, j} \bigr) \approx \mathbf{I} + \widetilde{\boldsymbol{\theta}}_{k}^{j, j} +\Delta t \widetilde{ \boldsymbol{\omega}}_{k}^{j, j}. $$

Since the vector $\boldsymbol{\omega}_{k}^{j,j}$ defines the local angular velocity at $t= t_{0}$, Eq. (4.4) holds for small $\Delta t$. With this relation, it follows that the nodal rotations $\boldsymbol{\theta}_{k}^{j,j}$ at $t= t_{0}$ can be derived from the rotation matrix as

4.5 $$ \widetilde{\boldsymbol{\theta}}_{k}^{j, j} \approx \frac{1}{2} \bigl( \mathbf{R}_{k}^{j} - \mathbf{R}_{k}^{j T} \bigr). $$

Moreover, the time derivative of $\boldsymbol{\theta}_{k}^{j,j}$ at $t= t_{0}$ is approximately the angular velocity vector $\boldsymbol{\omega}_{k}^{j,j}$. This gives a relation between the relative velocity and the generalized coordinates corresponding to the Craig–Bampton modes:

4.6 $$ \dot{\mathbf{q}}_{k}^{j, j} = \left [ \textstyle\begin{array}{c} \dot{\boldsymbol{\theta}}_{k}^{j, j}\\ \dot{\mathbf{u}}_{k}^{j, j} \end{array}\displaystyle \right ] \approx \left [ \textstyle\begin{array}{c} \boldsymbol{\omega}_{k}^{j,j}\\ \dot{\mathbf{u}}_{k}^{j, j} \end{array}\displaystyle \right ] = \mathbf{v}_{k}^{j, j}. $$

This relation, in combination with (3.7) shows that it is possible to relate the relative velocity of a material point $\dot{\mathbf{q}}_{k}^{j, j}$ to the absolute velocities $\mathbf{v}_{k}^{O,O}$ and $\mathbf{v}_{j}^{O,O}$:

4.7 $$ \dot{\mathbf{q}}_{k}^{j, j} = \bigl[ \mathbf{R}_{O}^{j} \bigr] \mathbf{v}_{k}^{O,O} - \bigl[ - \tilde{ \mathbf{r}}_{k}^{j,j} \bigr] \bigl[ \mathbf{R}_{O}^{j} \bigr] \mathbf{v}_{j}^{O,O}. $$

Using a floating frame formulation, the dynamic equations contain the absolute coordinates of the floating frame, as well as the relative coordinates of the interface points. With result (4.7), it is possible to eliminate the local elastic velocities from the dynamic equations and replace them by the absolute velocities of the floating frame and interface points [18]. At this point, the bodies can be coupled directly and Lagrange multipliers are no longer required to enforce constraints.

Because in this work the floating frame is not located at an interface point, the Craig–Bampton modes can still describe six rigid body motions. As the rigid body motion is already described by the floating frame, including both makes the system singular. Six additional constraints should be imposed on the Craig–Bampton modes in order to remove this singularity. These additional constraints enable the possibility to express the motion of the floating frame in terms of the motion of the interface points $P_{k}$ only. This yields a multibody formulation solely in terms of the absolute coordinates of the interface points.

## Elimination of the floating frame from the kinematic description

**Fig. 4:**
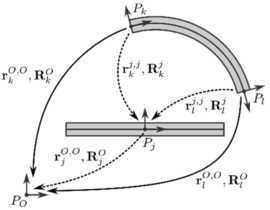
Local position of the interface coordinates in the floating frame (*dashed arrows*) and absolute position of the interface coordinates in the inertial frame (*solid arrows*). Figure was made using InkScape

Figure 4 shows a flexible body with floating frame $P_{j}$ and two interface points $P_{k}$ and $P_{l}$. The relative positions of the interface points with respect to the floating frame depend on the Craig–Bampton degrees of freedom $\mathbf{q}_{k}^{j,j}$ and $\mathbf{q}_{l}^{j,j}$. In the floating frame formulation, the equations of motion are expressed in terms of the absolute floating frame coordinates $\{ \mathbf{r}_{j}^{O,O}, \mathbf{R}_{j}^{O} \}$ and the relative coordinates $\mathbf{q}_{k}^{j,j}$ and $\mathbf{q}_{l}^{j,j}$ (dashed arrows). The idea is to reformulate the equations of motion by replacing these coordinates by the absolute motion of the interface points, defined by $\{ \mathbf{r}_{k}^{O,O}, \mathbf{R}_{k}^{O} \}$ and $\{ \mathbf{r}_{l}^{O,O}, \mathbf{R}_{l}^{O} \}$ (solid arrows).

Suppose that the body has $N$ interface points and that $P_{j}$ is not an interface point. The deformation of $P_{j}$ due to the Craig–Bampton modes related to interface point $P_{k}$ are collected in the ($6\times6$) modes matrix $\boldsymbol{\varPhi}_{k}^{j}$. The elastic deformation $\mathbf{q}_{j}^{j, j}$ in point $P_{j}$ is then a superposition of $\boldsymbol{\varPhi}_{k}^{j}$ and the generalized coordinates $\mathbf{q}_{k}^{j, j}$ in the interface points:

5.1 $$ \mathbf{q}_{j}^{j,j} = \sum_{k=1}^{N} \boldsymbol{\varPhi}_{k}^{j} \mathbf{q}_{k}^{j,j}. $$

In order to remove the 6 rigid body modes from the set of Craig–Bampton modes, the requirement is made that the elastic body has no elastic deformation in $P_{j}$. Unfortunately, since the relation between the relative position of the interface points and the absolute positions of the interface points and the floating frame is nonlinear, these additional constraints cannot be solved explicitly on the position level. However, this can be done on the velocity level. To this end, Eq. (4.7) is substituted in the time derivative of (5.1). It follows from the requirement of zero elastic deformation that this must also be zero:

5.2 $$ \dot{\mathbf{q}}_{j}^{j,j} = \sum _{k=1}^{N} \boldsymbol{\varPhi}_{k}^{j} \bigl( \bigl[ \mathbf{R}_{O}^{j} \bigr] \mathbf{v}_{k}^{O,O} - \bigl[ - \tilde{\mathbf{r}}_{k}^{j,j} \bigr] \bigl[ \mathbf{R}_{O}^{j} \bigr] \mathbf{v}_{j}^{O,O} \bigr) = \boldsymbol{0}. $$

Rewriting yields a relation between the velocity of the floating frame and the absolute velocities of the interface points:

5.3 $$ \bigl[ \mathbf{Q}^{j} \bigr] \bigl[ \mathbf{R}_{O}^{j} \bigr] \mathbf{v}_{j}^{O,O} = \sum _{k=1}^{N} \boldsymbol{\varPhi}_{k}^{j} \bigl[ \mathbf{R}_{O}^{j} \bigr] \mathbf{v}_{k}^{O,O}, \quad \quad \bigl[ \mathbf{Q}^{j} \bigr] \equiv \sum _{k=1}^{N} \boldsymbol{\varPhi}_{k}^{j} \bigl[ - \tilde{\mathbf{r}}_{k}^{j,j} \bigr]. $$

The ($6\times6$) matrix $[ \mathbf{Q}^{j} ]$ consists of 6 elastic displacement vectors defined by the sum of the Craig–Bampton modes multiplied with the local positions $[ - \tilde{\mathbf{r}}_{k}^{j, j} ]$ of the interface points. The matrices $[ - \tilde{\mathbf{r}}_{k}^{j, j} ]$ define the 6 rigid body modes of the deformed body in the interface points calculated relative to $P_{j}$. When the body is undeformed, the position vectors $\mathbf{r}_{k}^{j, j} $ equal the position vectors $\mathbf{x}_{k}^{j, j}$ and the resulting displacements in $[ \mathbf{Q}^{j} ]$ become unit displacements and so it equals the unity matrix $\mathbf{I}$. Since $\mathbf{r}_{k}^{j, j}$ equals $\mathbf{x}_{k}^{j, j}$ on leading order, $[ \mathbf{Q}^{j} ]$ can be assumed regular in all cases and can be inverted. Hence, (5.3) can be solved for the absolute velocity of the floating frame:

5.4 $$ \mathbf{v}_{j}^{O,O} = \bigl[ \mathbf{R}_{j}^{O} \bigr] \bigl[ \mathbf{Q}^{j} \bigr]^{-1} \sum _{k=1}^{N} \boldsymbol{\varPhi}_{k}^{j} \bigl[ \mathbf{R}_{O}^{j} \bigr] \mathbf{v}_{k}^{O,O}. $$

This can be written in compact matrix–vector notation as follows:

5.5 $$ \mathbf{v}_{j}^{O,O} = \bigl[ \mathbf{R}_{j}^{O} \bigr] [ \mathbf{Z}_{j} ] \bigl[ \overline{\mathbf{R}}_{O}^{j} \bigr] \mathbf{v}^{O,O}. $$

Here the ($6N\times 1$) vector $\mathbf{v}^{O,O}$ contains the absolute velocities of all interface points and the ($6N\times 6N$) rotation matrix $[ \overline{\mathbf{R}}_{O}^{j} ]$ is assembled from the ($6\times6$) rotation matrix $[ \mathbf{R}_{O}^{j} ]$:

5.6 $$ \mathbf{v}^{O, O} \boldsymbol{\equiv} \left [ \textstyle\begin{array}{c} \mathbf{v}_{1}^{O, O}\\ \vdots\\ \mathbf{v}_{N}^{O, O} \end{array}\displaystyle \right ],\quad \quad \bigl[ \overline{\mathbf{R}}_{O}^{j} \bigr] \equiv \left [ \textstyle\begin{array}{c@{\quad}c@{\quad}c} [ \mathbf{R}_{O}^{j} ] & & \\ & \ddots & \\ & & [ \mathbf{R}_{O}^{j} ] \end{array}\displaystyle \right ]. $$

Moreover, the ($6\times 6N$) matrix $[ \mathbf{Z}^{j} ]$ is defined using a ($6\times 6N$) matrix $[ \boldsymbol{\varPhi}_{CB}^{j} ]$ as follows:

5.7 $$ \bigl[ \mathbf{Z}^{j} \bigr] \equiv \bigl[ \mathbf{Q}^{j} \bigr]^{-1} \bigl[ \boldsymbol{\varPhi}_{CB}^{j} \bigr],\quad \quad \bigl[ \boldsymbol{\varPhi}_{CB}^{j} \bigr] \equiv \left [ \textstyle\begin{array}{c@{\quad}c@{\quad}c} \boldsymbol{\varPhi}_{1}^{j} & \ldots & \boldsymbol{\varPhi}_{N}^{j} \end{array}\displaystyle \right ]. $$

Upon substitution of (5.5) into (4.7), it is possible to express the local velocities of the deformed body in terms of the absolute velocities of the interface points, which can be written compactly as

5.8 $$ \dot{\mathbf{q}}^{j,j} = \left [ \textstyle\begin{array}{c@{\quad}c} [ \boldsymbol{\varPhi}_{\mathit{rig}}^{j} ] & \mathbf{I} \end{array}\displaystyle \right ] \left [ \textstyle\begin{array}{c} - [ \mathbf{Z}^{j} ]\\ \mathbf{I} \end{array}\displaystyle \right ] \bigl[ \overline{\mathbf{R}}_{O}^{j} \bigr] \mathbf{v}^{O,O}. $$

Here the ($6N\times1$) vector $\dot{\mathbf{q}}^{j,j}$ contains the relative velocities of all interface points due to elastic deformations of the body expressed in the floating frame and the ($6N\times6$) matrix $[ \boldsymbol{\varPhi}_{\mathit{rig}}^{j} ]$, which is the matrix of rigid body modes, is defined as

5.9 $$ \dot{\mathbf{q}}^{j,j} \boldsymbol{\equiv} \left [ \textstyle\begin{array}{c} \dot{\mathbf{q}}_{1}^{j,j}\\ \vdots\\ \dot{\mathbf{q}}_{N}^{j,j} \end{array}\displaystyle \right ],\quad \quad \bigl[ \boldsymbol{\varPhi}_{\mathit{rig}}^{j} \bigr] \equiv \left [ \textstyle\begin{array}{c} [ - \tilde{\mathbf{r}}_{1}^{j,j} ]\\ \vdots\\ {} [ - \tilde{\mathbf{r}}_{N}^{j,j} ] \end{array}\displaystyle \right ]. $$

In short, (5.8) can be written as

5.10 $$ \dot{\mathbf{q}}^{j,j} = \bigl[ \mathbf{T}^{j} \bigr] \bigl[ \overline{\mathbf{R}}_{O}^{j} \bigr] \mathbf{v}^{O,O}, \quad \quad \bigl[ \mathbf{T}^{j} \bigr] \equiv \left [ \textstyle\begin{array}{c@{\quad}c} [ \boldsymbol{\varPhi}_{\mathit{rig}}^{j} ] & \mathbf{I} \end{array}\displaystyle \right ] \left [ \textstyle\begin{array}{c} - [ \mathbf{Z}^{j} ]\\ \mathbf{I} \end{array}\displaystyle \right ] = \mathbf{I} - \bigl[ \boldsymbol{\varPhi}_{\mathit{rig}}^{j} \bigr] \bigl[ \mathbf{Z}^{j} \bigr]. $$

The combination of Eqs. (5.5) and (5.10) makes it possible to rewrite the dynamic equations of motion in floating frame formulation to a formulation in terms of the inertial frame formulation. This is demonstrated in the next section.

## The equations of motion in absolute interface coordinates

The derivation of the equations of motion in terms of the absolute interface coordinates are derived based on the equations of motion in the floating frame formulation. For each flexible body in a multibody system, the standard equations of motion can be written as

6.1 $$ \mathbf{M} \ddot{\mathbf{q}} + \mathbf{Q}_{v} + \mathbf{Kq} = \mathbf{Q}_{e} + \mathbf{Q}_{c}. $$

Here $\mathbf{M}$ and $\mathbf{K}$ are the mass and stiffness matrices, respectively. The vector of generalized coordinates $\mathbf{q}$ is the set of the floating frame coordinates and the local elastic coordinates corresponding to the Craig–Bampton modes. $\mathbf{Q}_{v}$ is the vector of quadratic velocity inertia forces, $\mathbf{Q}_{e}$ is the vector of externally applied forces and $\mathbf{Q}_{c}$ is the vector of constraint forces. A detailed derivation of Eq. (6.1), based on the principle of virtual work, can be found in standard textbooks on multibody dynamics, e.g., [12]. Equation (6.1) can be partitioned as follows:

6.2 $$ \left [ \textstyle\begin{array}{c@{\quad}c} \mathbf{M}_{rr} & \mathbf{M}_{rf}\\ \mathbf{M}_{fr} & \mathbf{M}_{ff} \end{array}\displaystyle \right ] \left [ \textstyle\begin{array}{c} \mathbf{a}_{j}^{O,O}\\ \ddot{\mathbf{q}}^{j,j} \end{array}\displaystyle \right ] + \left [ \textstyle\begin{array}{c} \mathbf{Q}_{v,r}\\ \mathbf{Q}_{v,f} \end{array}\displaystyle \right ] + \left [ \textstyle\begin{array}{c@{\quad}c} \boldsymbol{0} & \boldsymbol{0}\\ \boldsymbol{0} & \mathbf{K}_{ff} \end{array}\displaystyle \right ] \left [ \textstyle\begin{array}{c} \boldsymbol{0}\\ \mathbf{q}^{j,j} \end{array}\displaystyle \right ] = \left [ \textstyle\begin{array}{c} \mathbf{Q}_{e,r}\\ \mathbf{Q}_{e,f} \end{array}\displaystyle \right ] + \left [ \textstyle\begin{array}{c} \mathbf{Q}_{c,r}\\ \mathbf{Q}_{c,f} \end{array}\displaystyle \right ]. $$

Here the subscripts $r$ and $f$ refer to the rigid and flexible partitions of the matrices and vectors, respectively. $\mathbf{a}_{j}^{O,O}$ is the absolute acceleration of the floating frame. The matrices $\mathbf{M}_{ff}$ and $\mathbf{K}_{ff}$ are directly obtained from the linear finite element model of the body on which the Craig–Bampton reduction is applied.

In order to rewrite Eq. (6.2) in terms of the absolute interface coordinates, a coordinate transformation is used. On the velocity level, this coordinate transformation is obtained from combining Eqs. (5.5) and (5.10):

6.3 $$ \left [ \textstyle\begin{array}{c} \mathbf{v}_{j}^{O,O}\\ \dot{\mathbf{q}}^{j,j} \end{array}\displaystyle \right ] = \left [ \textstyle\begin{array}{c} [ \mathbf{R}_{j}^{O} ] [ \mathbf{Z}^{j} ] [ \overline{\mathbf{R}}_{O}^{j} ]\\ {} [ \mathbf{T}^{j} ] [ \overline{\mathbf{R}}_{O}^{j} ] \end{array}\displaystyle \right ] \mathbf{v}^{O,O} = \mathbf{A v}^{O,O}, $$

where $\mathbf{A}$ shortly denotes the coordinate transformation matrix. Differentiating (6.3) with respect to time yields the coordinate transformation on the acceleration level. By means of the product rule, this transformation consists of a transformation of the accelerations themselves and a squared velocity term containing the time derivative of the transformation matrix:

6.4 $$ \left [ \textstyle\begin{array}{c} \mathbf{a}_{j}^{O,O}\\ \ddot{\mathbf{q}}^{j,j} \end{array}\displaystyle \right ] = \mathbf{A a}^{O,O} + \dot{\mathbf{A}} \mathbf{v}^{O,O}. $$

Substituting this coordinate transformation into (6.2) yields the equation of motion in terms of the absolute interface coordinates. By direct computation of the matrix multiplications concerned with this coordinate transformation, it is found that the resulting equation of motion can be written in the following form:

6.5 $$ \bigl[ \overline{\mathbf{R}}_{j}^{O} \bigr] [ \mathbf{M}_{ff} ] \bigl[ \overline{\mathbf{R}}_{O}^{j} \bigr] \mathbf{a}^{O,O} + \mathbf{Q}_{v}^{O} + \bigl[ \overline{\mathbf{R}}_{j}^{O} \bigr] \bigl[ \mathbf{T}^{j} \bigr]^{T} [ \mathbf{K}_{ff} ] \mathbf{q}^{j,j} = \mathbf{Q}_{j}^{O}. $$

Here the main inertia term can be recognized as the body’s local Craig–Bampton mass matrix rotated to the global frame multiplied with the absolute accelerations of the interface points. Other inertia forces that result from the coordinate transformation (6.4) are quadratic in the velocity and are therefore combined with the quadratic velocity inertia terms in the original equation of motion and represented by $\mathbf{Q}_{v}^{O}$. This term is not taken into account in standard corotational formulations. The procedure as explained here could be used to include these additional inertia effects in the corotational formulation as well. However, taking this term into account is only significant in case of systems operating at high velocities. In the elastic term, the Craig–Bampton stiffness matrix is multiplied with the local elastic coordinates. The pre-multiplication with $[ \mathbf{T}^{j} ]^{T}$ can be interpreted as an operation that extracts the elastic deformations from the absolute interface coordinates by eliminating the rigid body component, see also the definition of $[ \mathbf{T}^{j} ]$ in Eq. (5.10). The resulting elastic forces are transformed to the global frame by the rotation matrix. $\mathbf{Q}_{j}^{O}$ contain the forces applied on the interface points expressed in the global frame. The constraint forces are also included simply in $\mathbf{Q}_{j}^{O}$, because the constraints are enforced at the location of the interface points.

As explained in Sect. 5, it is not possible to construct an explicit coordinate transformation between the floating frame coordinates, local interface coordinates and absolute interface coordinates on the position level. Hence, due to the elastic term the equation of motion (6.5) is not entirely formulated in terms of the absolute interface coordinates. However, when numerically integrating in time, the equation of motion does not need to be solved for the large absolute position of the interface points. Instead, it is only solved for the small increment in the interface coordinates that occurs during the time increment. The time-discretized equations are linear in this position increment and tangent to the current configuration space. For that reason, the same coordinate transformation matrix as in (6.3) can be applied on this position increment. In this way, the problem that needs to be solved at every time step is formulated completely in terms of the absolute interface coordinates.

The location of the floating frame at the next time step can be determined from integrating (5.5). Theoretically, however, the numerical error may cause the floating frame to drift. For that reason, the location of the floating frame is determined from the absolute interface coordinates on the position level using a Newton–Raphson procedure. As an initial estimate, the floating frame position of the current time step is used. In practice, only few Newton–Raphson iterations are required as the time steps are sufficiently small. With the absolute position of the interface coordinates and the floating frame determined, the local elastic deformation can be determined directly.

The presented method can also be used to solve large deformation, static problems. By simplifying (6.5) for this case, the equilibrium equations for one body are obtained:

6.6 $$ \bigl[ \overline{\mathbf{R}}_{j}^{O} \bigr] [ \mathbf{T}_{j} ]^{T} [ \mathbf{K}_{j} ] \mathbf{q}^{j,j} = \mathbf{Q}_{j}^{O}. $$

These equations are nonlinear in the elastic deformations $\mathbf{q}^{j,j}$ as both $[ \overline{\mathbf{R}}_{j}^{O} ]$ and $[ \mathbf{T}_{j} ]$ depend on them. However, these equations can be solved incrementally using a standard updated Lagrangian approach. In this way, equations are obtained in terms of the position increment, which can be rewritten in terms of the absolute interface coordinates, similarly as discussed for the dynamic case.

## Validation

In order to validate the method presented in this work, its solution to four problems is compared with that of other standard software. These validation problems consist of a static cantilever beam subjected to a large vertical tip force, a 2D and 3D slider–crank mechanism with a flexible connector and a 3D spinning beam on a spherical joint. The solution of the static problem is compared with Spacar and Ansys. The solutions of the dynamic problems are compared with Spacar and MSC/Adams. Spacar is a finite element based multibody software that uses the corotational formulation [19]. Ansys uses an inertial frame formulation for their nonlinear finite element analyses. MSC/Adams uses a floating frame of reference formulation.

**Fig. 5:**
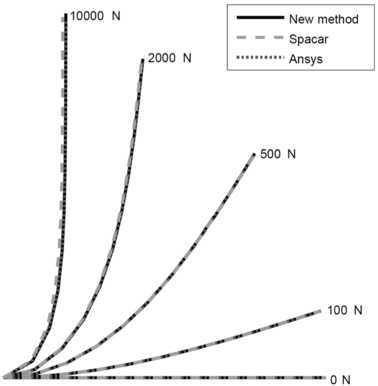
Deflection of a cantilever beam subjected to a tip force compared with Spacar and Ansys. Figure was made using Matlab for plotting and Adobe Illustrator for labels

For the static problem, a cantilever beam with circular cross section was modeled with 10 bodies. The total length of the beam is 1 m. The outer radius of the cross-section is 0.01 m has a wall thickness of 0.001 m. The Young’s modulus is 70.0E9 Pa. The beam was incrementally loaded starting at 100 N and increasing to 10000 N. Results have been obtained with the new method, Spacar and Ansys. The computed deformed beam shapes are shown in Fig. 5 for applied loads of 100, 500, 2000, and 10000 N. The figures show good agreement between the new code and both Spacar and Ansys.

**Fig. 6:**
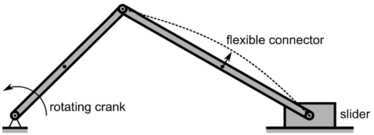
2D Slider–crank mechanism with flexible connector. Figure was made using InkScape

The dynamic 2D slider–crank problem is adopted from [20] and shown in Fig. 6. The rigid crank with length of 0.15 m is rotating with a constant angular velocity of 150 rad/s. The flexible connector with length of 0.3 m has a uniform circular cross-section with a diameter of 0.006 m and is made of steel. In the simulation a Young’s modulus of 0.2E12 Pa and a mass density of $7.87\mbox{E}3~\mbox{kg}/\mbox{m}^{3}$ is used. The end of the connector is connected to a slider with a mass half the mass of the connector. The slider is able to translate without friction on its base.

The angular velocity of the crank introduces an initial linear velocity and an angular velocity of the connector, assuming no deformation. The connector is modeled with two bodies. Hence, this model has three nodes: the two interface nodes and a half-way node at the location of the floating frame.

**Fig. 7:**
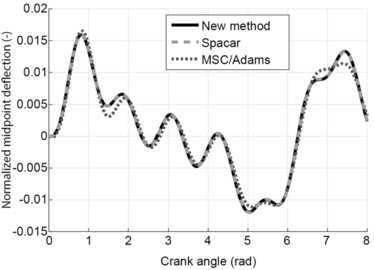
Mid-point deformation of the connector compared with Spacar and MSC/Adams. Figure was made using Matlab for plotting and Adobe Illustrator for labels

As output the displacement of the midpoint perpendicular to the undeformed beam was determined and plotted against the crank angle. The results are shown in Fig. 7. This figure also shows the results obtained with Spacar in which longitudinal deformations due to normal forces are suppressed. Moreover, results obtained with MSC/Adams are included. As Fig. 7 shows, the new method agrees very well with the results obtained with Spacar. The results obtained with MSC/Adams show small differences.

**Fig. 8:**
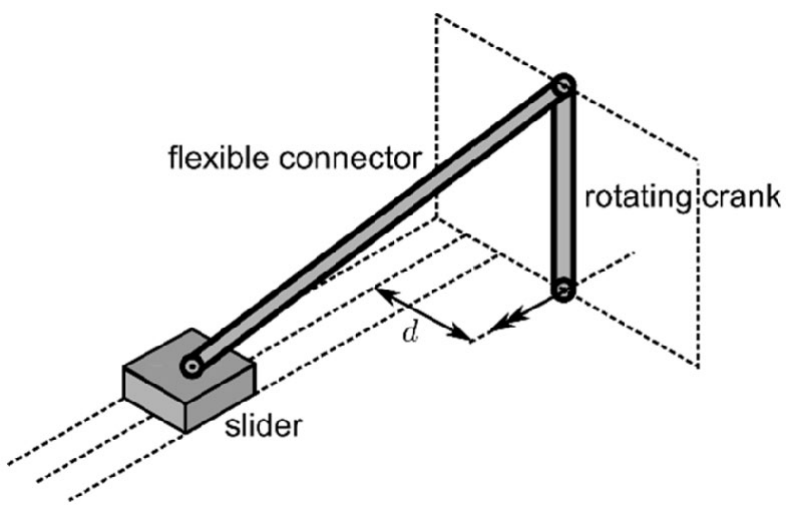
3D Slider–crank mechanism with flexible connector. Figure was made using InkScape

The dynamic 3D slider–crank mechanism is adopted from [21] and shown in Fig. 8. The physical properties of the mechanism are the same as in the 2D case described above. The horizontal position of the rotation axis $d$ is 0.1 m. In the initial configuration, the crank is oriented vertically upward.

**Fig. 9:**
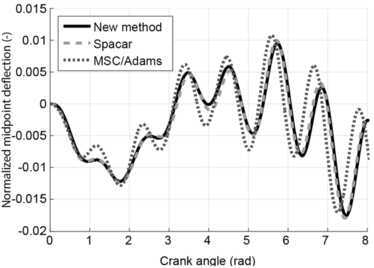
Mid-point deformation of the connector compared with Spacar and MSC/Adams. Figure was made using Matlab for plotting and Adobe Illustrator for labels

As output the displacement of the midpoint in its local $y$-direction was determined and plotted against the crank angle. The results are shown in Fig. 9. It can be seen that also in this case the results obtained with the new method are very close to the results obtained with Spacar. The results obtained with MSC/Adams again show a small difference in comparison with the other two methods.

**Fig. 10:**
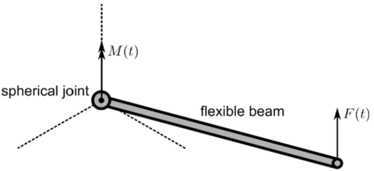
Flexible beam on a spherical joint. Figure was made using InkScape

The 3D spinning beam on a spherical joint is adopted from [14] and shown in Fig. 10. The physical properties, prescribed loads and simulation settings are the same as described in this reference: The beam has length 141.42 mm, cross-section $9.0~\mbox{mm}^{2}$ and moment of inertia $6.75~\mbox{mm}^{4}$. The material has a mass density $7.8\mbox{E-}3~\mbox{kg}/\mbox{mm}^{-3}$ and Young’s Modulus $2.1\mbox{E}6~\mbox{N}/\mbox{mm}^{2}$. The beam is modeled with two bodies. A torque of 200 N mm is applied about the vertical axis during the first 10.2 seconds. After 15 seconds, an impulsive vertical tip force of 100 N is applied.

**Fig. 11:**
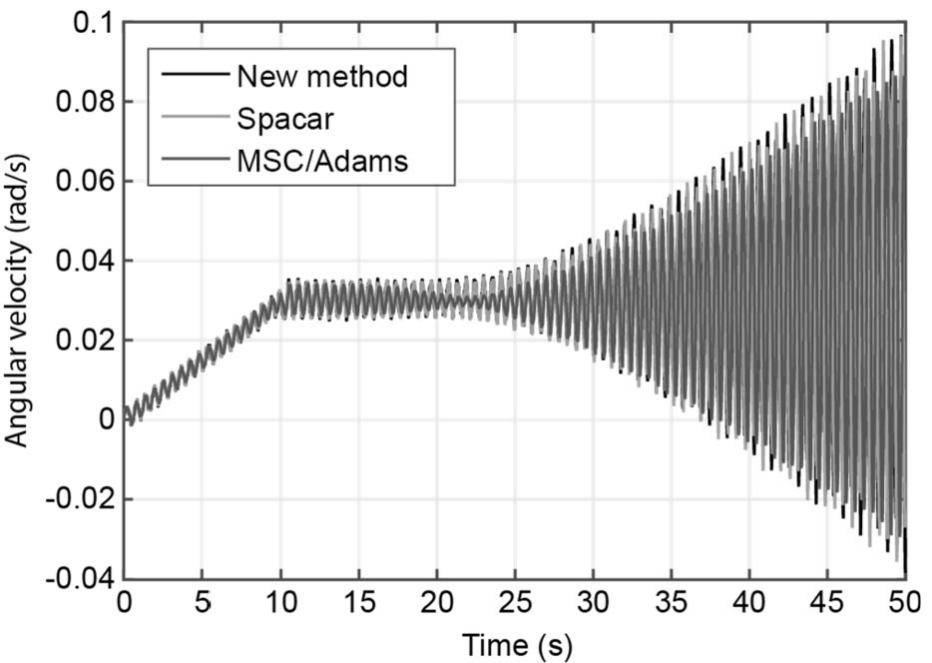
Angular velocity about the vertical axis at the base of the beam compared with Spacar and MSC/Adams. Figure was made using Matlab for plotting and Adobe Illustrator for labels

As output the absolute angular velocity about the vertical axis at the base of the beam was determined and plotted as a function of time. The results are shown in Fig. 11. It was observed in [14] that different methods show different results only after the impulsive vertical force is applied. In Fig. 11 it can be seen that all methods used here produce very similar results even after this moment. Comparing all results with those published in [14], it can be seen that the new method presented in this work is most comparable to results of the nonlinear finite element formulation used in [14].

## Conclusion

Describing the kinematics of a flexible multibody system comes down to the kinematic formulation of the motion of the interface points. In the inertial frame and corotational frame formulations, the absolute interface coordinates are part of the degrees of freedom, allowing for a direct application of the constraints. This is in contrast to the floating frame formulation, which requires the use of Lagrange multipliers. In this work, it has been demonstrated that the absolute floating frame coordinates and the local elastic coordinates that appear in the equation of motion of a floating frame formulation can be replaced by the absolute interface coordinates. Consequently, the new method does not require Lagrange multipliers to enforce the kinematic constraints. In this way a floating frame formulation in terms of a minimal set of coordinates is successfully obtained. Validation of the method with static and dynamic problem found in literature has shown to yield reliable results in all cases.

The use of Craig–Bampton modes as local shape functions is crucial in the presented procedure. The rigid body motions contained in these Craig–Bampton modes are employed to eliminate the floating frame coordinates from the system. The problem of establishing the kinematic coordinate transformation presented in this work for the floating frame formulation is in fact comparable to the problem of expressing the corotational frame coordinates in terms of the element’s absolute nodal coordinates for a corotational frame formulation. Only here this problem is encountered at the level of an entire body instead of on the level of a single element. An advantage of the new method is that it can be applied to systems that consist of arbitrarily shaped three-dimensional bodies that have an arbitrary number of interface points. Also in this sense, it can be seen as a generalization of the corotational frame formulation, which is only developed for a limited number of parameterized finite elements, such as beams, plates and shells.

As such, this paper not only provides valuable insight in the relations between different multibody formulations, but it also offers the possibility to reduce geometric nonlinear systems by applying important modal order reduction techniques in a body’s local frame. This is found a convenient way of creating so-called superelements in a flexible multibody formulation. The use of the term superelement emphasizes the striking similarity between the floating frame and corotational frame formulations at this point. For each flexible body, the tangent mass and stiffness matrices, reduced to the interface points can be obtained from detailed linear finite element models. These system matrices can directly be applied in the flexible multibody analysis of the entire system.
